# Believing and Appraising in Context: Cognizing Experiences as Events

**DOI:** 10.3389/fpsyg.2022.913603

**Published:** 2022-05-19

**Authors:** Ann Taves, Raymond F. Paloutzian

**Affiliations:** ^1^Department of Religious Studies, University of California, Santa Barbara, Santa Barbara, CA, United States; ^2^Department of Psychology, Westmont College, Santa Barbara, CA, United States

**Keywords:** belief, believing, credition, event, event cognition, meaning making

It is clear that schemas and expectations unconsciously affect how people interpret their experience in real time (van Elk and Aleman, 2017; Van Leeuwen and van Elk, 2019). It is also clear that cultural beliefs as manifest in worldviews and ways of life affect how people consciously reflect on and reappraise their experiences (Kelly, 1955; Saroglou and Cohen, 2011, 2013). How these two processes are linked is not so clear. Here we argue that event cognition not only supplies a promising bridge between unconscious and conscious information processing, but allows us to further integrate research on unconscious appraisal processes (Scherer et al., 2001), conscious attributions (Kelley and Michela, 1980; Malle, 1999, 2004), and multi-level approaches to meaning making (Park and Folkman, 1997; Park, 2010; Markman et al., 2013) and believing (for an overview, see Seitz and Angel, 2020)^1^.

## What Is an Event?

The *Oxford English Dictionary* defines *events* simply as things that happen. Philosophers tend to begin with this general definition when discussing events, noting that “this definition merely shifts the burden [of definition] to clarifying the meaning of ‘happen”’ (Casati and Varzi, 2020). For scientific purposes, it is important to distinguish between things that happen regardless of whether there is a living organism to perceive them and happenings that living organisms perceive. Molecules move and stars collide regardless of whether living organisms are present. In doing so happenings generate information. Because far more information is generated than any organism can perceive and process, organisms have evolved to perceive and process the information that they need to survive. Humans and some other animals do so by segmenting information, that is, by a cognitive process that divides it into chunks with a beginning and an end (Baldwin and Kosie, 2021; Ross and Easton, 2022).

Researchers who study this cognitive process define an event more technically as “a segment of time at a given location conceived by an observer to have a beginning and an end” (Zacks and Tversky, 2001, p. 3). This means that an event is spatially and temporally located and is perceived from the point of view of the subject. An event is constructed as the brain chunks the dynamic flow of incoming information into segments. Most researchers also agree that the extracted segment is perceived as coherent and causally related, which points to the basis on which we chunk the flow of information (Hohwy et al., 2021).

The cognitive definition of an event has implications for how we think about experience (an uncountable noun) and experiences (a plural noun). The former designates the flow of information of which we are aware. The latter are self-reported events that have a beginning and an end; this means that from a cognitive perspective, experiences are events regardless of whether they are externally verifiable or not (for fuller discussion, see Taves and Asprem, 2017). Treating experiences as events allows us to investigate how the flow of information of which we are aware (experience) is transformed cognitively into structured units (experiences) with a beginning and end that we can describe, remember, and recount if and when we attend to them. Doing so allows us to link subpersonal and personal levels of analysis and integrate several lines of research in psychology, including multi-level approaches to meaning making and believing.

What then is not an event? Although philosophers debate this issue, they generally agree that physical objects in isolation are not events and that events occur when things change or interact (Shipley, 2008; Casati and Varzi, 2020). If we extend the criterion of change to mental things, such as ideas, beliefs, concepts, and goals, then beliefs all by themselves are not events. Events occur when beliefs change or interact, i.e., in the process of believing. How then do we cognize events?

## Working Models as Probabilistic Representations

Most researchers understand the processes by which events are initially cognized within a predictive processing framework (Hohwy et al., 2021).

Bottom-up input is weighed against a top-down prediction of what is happening based on prior experience. The prediction of what is happening is represented in a working model of an event. The working model is, thus, is a probabilistic assessment (i.e., appraisal) of incoming information in light of prior experience. The model is retained as long as it more or less fits with the incoming information. If there is a significant change in the input, an error signal is generated, which leads to a new or revised event model (Radvansky and Zacks, 2014, 2017; Zacks, 2020). The process of assessing the incoming information can be viewed as a meaning making process and the probabilistic assessment as an appraisal. The working model that is generated based on this assessment is a representation of the event. It can also be viewed as an implicit belief regarding what happened (Seitz et al., 2017, 2018; Paloutzian et al., 2021).

The working model is generated in working memory and thus is fleeting (like the dream you can't remember) unless transferred to long-term memory (Zacks, 2020, pp. 172–177). If the event is stored in long-term memory, we can remember it, narrate it, and reflect on it. In other words, the working model links what we consciously experience as happening with underlying cognitive processes and, if retained in long-term memory, allows us to recall and reflect on past events. Each time we recall an event, we construct a new event model in working memory with its own new spatio-temporal context.

In sum, the sensory input from the body, environment, and prior experience interact to form a working model of what is happening. Insofar as we are conscious of the contents of working memory, we are conscious of the contents of the working model. That is how we experience the event and come to believe—at least implicitly—that an event occurred (Seitz et al., 2022). If our experience is stored in long-term memory, we can remember and recount it. But these are separate events with their own working models of what is happening.

## Working Models and Prior Experience

The working model is based on a probabilistic assessment of incoming information *in light of prior experience* (Zacks, 2020, pp. 177–180). In psychological terms, the brain assesses incoming information in light of schemas and expectations. Schemas and expectations, although likely built on shared, reliably developing templates, are typically elaborated in culturally specific ways and acquired through cultural learning. Each component of a working model—time, space, objects, sensations, relations, and causes—draws on prior experience. With respect to time, schemas provide an expected time frame for the event. With respect to space, schemas identify the specific place or type of space in which the event is occurring. Schemas and expectations determine the types of agents—visible and invisible—that may be involved, identify who is involved, what they are doing and why. They allow us to recognize the objects involved and assess what is happening to them. They allow us to identify what we are sensing or feeling. Finally, overall event schemas help us understand how all these things are related and what is causing it to happen. Because working models rely on prior experience, we would expect infants, foreigners, and experts to have representations of an event that differ from those of the average culturally literate adult.

## Evolved and Cultural Knowledge

Although people's representations of an event differ based on their prior knowledge, human's evolved capacity to chunk the flow of information into events means that everyone—including infants—can recognize that something has happened. Thus, there is growing evidence that infants can attend to structured patterns in goal-directed activity and that these patterns provide a basic sense of where to segment the dynamic flow (Levine et al., 2019; Zacks, 2020). When we confront a new situation, we draw on those basic capabilities. Then, as we grow and develop in a particular time and place, we become able to comprehend and recall events with greater ease and accuracy. We become more “fluent.” We learn to pick out the relevant details of events that allow us to efficiently predict what is happening and guide us in deciding what to do. We do so in the context of particular culturally distinct ways of life. The event models of fluent adults are culture specific; they include appraisals and beliefs guided by and adapted to the culture in which one is imbedded (Baldwin and Kosie, 2021).

To illustrate, imagine a cultural event such as going to a Catholic Mass. A young child would understand that the event had a beginning and an end and contained any number of subevents, such as entering and leaving the church, walking to the front the church with their parents, and returning to their seats. When a bell was rung, the child's attention might be drawn to the altar and the man who is doing something there. An adult who knew nothing of Catholic ritual would also recognize these and other subevents. If they had experienced other rituals, they would likely recognize it as such, without knowing much else. Culturally fluent Catholics, however, would have internalized an event model of the Mass as a ritual that recapitulates Jesus' death and resurrection and his promise to be present in the sharing of bread and wine. They would know that the words of the priest over the bread and wine make Christ present and, thus, why the bell draws their attention to that point in the service.

## Re-Appraising Events

Events, as they initially surface to awareness, can take many forms. Most are routine; we give them little thought. Some events, however, stand out because they are puzzling, surprising, disturbing, or life-changing. These are the events we tend to remember, recount, and in some cases reappraise. If an experience does not fit with what we have learned to expect or believe, we return to it to try to figure out what happened or how to cope with what we know or believe happened. This process of making meaning out of ambiguity, appraising it, remaking meaning, reappraisal, and so on has been well researched and documented as a series of events (Park and Folkman, 1997; Park, 2010; Markman et al., 2013).

Understanding the meaning making process as a series of events, therefore, implies that the initial appraisal of meaning takes place as part of the initial event. This generates what Park (2010) refers to as the “situational” meaning of the event. But when someone consciously assesses their initial sense of what happened in light of their overall set of beliefs and goals, that constitutes a subsequent event, and the processes of coping with discrepancies between the situational and global meanings generates a whole series of additional events with their modified beliefs.

The working model of what is happening now, thus, allows us to think in two directions. We can (1) think about change over time as an initial event that is reappraised in the context of subsequent events, or we can (2) think about the levels of processing that give rise to an event model in the context of a single event. The first is an explicit reflective cultural process; the second relies on culturally learned expectations and schemas that function as priors in the probabilistic assessment of what is happening at any given moment. Treating experiences as events allows us to consider the components that interact to generate an experience and compare the interaction of schemas and expectations with phenomenological features of experiences in a variety of different cultural contexts (for a full discussion, see Taves and Barlev, 2022).

## Author Contributions

AT conceptualized the concept of events and how minds cognize events as related to the chunking and perception of things happening in real time. RP added their relationship to the processes of believing. Both authors contributed to the article and approved the submitted version.

## Conflict of Interest

The authors declare that the research was conducted in the absence of any commercial or financial relationships that could be construed as a potential conflict of interest.

## Publisher's Note

All claims expressed in this article are solely those of the authors and do not necessarily represent those of their affiliated organizations, or those of the publisher, the editors and the reviewers. Any product that may be evaluated in this article, or claim that may be made by its manufacturer, is not guaranteed or endorsed by the publisher.

## Footnotes

^1^Psychological interest in events emerged within both clinical and experimental psychology to produce lines of research that focus on differences levels of analysis. In clinical psychology, Kelly's (1955) personal construct theory, which is centrally concerned with the role of constructs in the interpretation of events, was influenced by hermeneutic approaches in the humanities (Butt and Warren, 2016). In experimental psychology, the focus has been on the perceptual processes (i.e., mechanisms) involved in cognizing events (Shipley, 2008; Zacks, 2020).
